# Monitoring the Impact of Influenza by Age: Emergency Department Fever and Respiratory Complaint Surveillance in New York City

**DOI:** 10.1371/journal.pmed.0040247

**Published:** 2007-08-07

**Authors:** Donald R Olson, Richard T Heffernan, Marc Paladini, Kevin Konty, Don Weiss, Farzad Mostashari

**Affiliations:** New York City Department of Health and Mental Hygiene, New York, New York, United States of America; Imperial College London, United Kingdom

## Abstract

**Background:**

The importance of understanding age when estimating the impact of influenza on hospitalizations and deaths has been well described, yet existing surveillance systems have not made adequate use of age-specific data. Monitoring influenza-related morbidity using electronic health data may provide timely and detailed insight into the age-specific course, impact and epidemiology of seasonal drift and reassortment epidemic viruses. The purpose of this study was to evaluate the use of emergency department (ED) chief complaint data for measuring influenza-attributable morbidity by age and by predominant circulating virus.

**Methods and Findings:**

We analyzed electronically reported ED fever and respiratory chief complaint and viral surveillance data in New York City (NYC) during the 2001–2002 through 2005–2006 influenza seasons, and inferred dominant circulating viruses from national surveillance reports. We estimated influenza-attributable impact as observed visits in excess of a model-predicted baseline during influenza periods, and epidemic timing by threshold and cross correlation. We found excess fever and respiratory ED visits occurred predominantly among school-aged children (8.5 excess ED visits per 1,000 children aged 5–17 y) with little or no impact on adults during the early-2002 B/Victoria-lineage epidemic; increased fever and respiratory ED visits among children younger than 5 y during respiratory syncytial virus-predominant periods preceding epidemic influenza; and excess ED visits across all ages during the 2003–2004 (9.2 excess visits per 1,000 population) and 2004–2005 (5.2 excess visits per 1,000 population) A/H3N2 Fujian-lineage epidemics, with the relative impact shifted within and between seasons from younger to older ages. During each influenza epidemic period in the study, ED visits were increased among school-aged children, and each epidemic peaked among school-aged children before other impacted age groups.

**Conclusions:**

Influenza-related morbidity in NYC was highly age- and strain-specific. The impact of reemerging B/Victoria-lineage influenza was focused primarily on school-aged children born since the virus was last widespread in the US, while epidemic A/Fujian-lineage influenza affected all age groups, consistent with a novel antigenic variant. The correspondence between predominant circulating viruses and excess ED visits, hospitalizations, and deaths shows that excess fever and respiratory ED visits provide a reliable surrogate measure of incident influenza-attributable morbidity. The highly age-specific impact of influenza by subtype and strain suggests that greater age detail be incorporated into ongoing surveillance. Influenza morbidity surveillance using electronic data currently available in many jurisdictions can provide timely and representative information about the age-specific epidemiology of circulating influenza viruses.

## Abstract 2

Don Olson and colleagues report that influenza-related morbidity in NYC from 2001 to 2006 was highly age- and strain-specific and conclude that surveillance using electronic data can provide timely and representative information about the epidemiology of circulating influenza viruses.

## Editors' Summary

**Background.:**

Seasonal outbreaks (epidemics) of influenza (a viral infection of the nose, throat, and airways) send millions of people to their beds every winter. Most recover quickly, but flu epidemics often disrupt daily life and can cause many deaths. Seasonal epidemics occur because influenza viruses continually make small changes to the viral proteins (antigens) that the human immune system recognizes. Consequently, an immune response that combats influenza one year may provide partial or no protection the following year. Occasionally, an influenza virus with large antigenic changes emerges that triggers an influenza pandemic, or global epidemic. To help prepare for both seasonal epidemics and pandemics, public-health officials monitor influenza-related illness and death, investigate unusual outbreaks of respiratory diseases, and characterize circulating strains of the influenza virus. While traditional influenza-related illness surveillance systems rely on relatively slow voluntary clinician reporting of cases with influenza-like illness symptoms, some jurisdictions have also started to use “syndromic” surveillance systems. These use electronic health-related data rather than clinical impression to track illness in the community. For example, increased visits to emergency departments for fever or respiratory (breathing) problems can provide an early warning of an influenza outbreak.

**Why Was This Study Done?:**

Rapid illness surveillance systems have been shown to detect flu outbreaks earlier than is possible through monitoring deaths from pneumonia or influenza. Increases in visits to emergency departments by children for fever or respiratory problems can provide an even earlier indicator. Researchers have not previously examined in detail how fever and respiratory problems by age group correlate with the predominant circulating respiratory viruses. Knowing details like this would help public-health officials detect and respond to influenza epidemics and pandemics. In this study, the researchers have used data collected between 2001 and 2006 in New York City emergency departments to investigate these aspects of syndromic surveillance for influenza.

**What Did the Researchers Do and Find?:**

The researchers analyzed emergency department visits categorized broadly into a fever and respiratory syndrome (which provides an estimate of the total visits attributable to influenza) or more narrowly into an influenza-like illness syndrome (which specifically indicates fever with cough and/or sore throat) with laboratory-confirmed influenza surveillance data. They found that emergency department visits were highest during peak influenza periods, and that the affect on different age groups varied depending on the predominant circulating viruses. In early 2002, an epidemic reemergence of B/Victoria-lineage influenza viruses caused increased visits among school-aged children, while adult visits did not increase. By contrast, during the 2003–2004 season, when the predominant virus was an A/H3N2 Fujian-lineage influenza virus, excess visits occurred in all age groups, though the relative increase was greatest and earliest among school-aged children. During periods of documented respiratory syncytial virus (RSV) circulation, increases in fever and respiratory emergency department visits occurred in children under five years of age regardless of influenza circulation. Finally, the researchers found that excess visits to emergency departments for fever and respiratory symptoms preceded deaths from pneumonia or influenza by about two weeks.

**What Do These Findings Mean?:**

These findings indicate that excess emergency department visits for fever and respiratory symptoms can provide a reliable and timely surrogate measure of illness due to influenza. They also provide new insights into how different influenza viruses affect people of different ages and how the timing and progression of each influenza season differs. These results, based on data collected over only five years in one city, might not be generalizable to other settings or years, warn the researchers. However, the present results strongly suggest that the routine monitoring of influenza might be improved by using electronic health-related data, such as emergency department visit data, and by examining it specifically by age group. Furthermore, by showing that school-aged children can be the first people to be affected by seasonal influenza, these results highlight the important role this age group plays in community-wide transmission of influenza, an observation that could influence the implementation of public-health strategies such as vaccination that aim to protect communities during influenza epidemics and pandemics.

**Additional Information.:**

Please access these Web sites via the online version of this summary at http://dx.doi.org/10.1371/journal.pmed.0040247.

• US Centers for Disease Control and Prevention provides information on influenza for patients and health professionals and on influenza surveillance in the US (in English, Spanish, and several other languages)

• World Health Organization has a fact sheet on influenza and on global surveillance for influenza (in English, Spanish, French, Russian, Arabic, and Chinese)

• The MedlinePlus encyclopedia contains a page on flu (in English and Spanish)

• US National Institute of Allergy and Infectious Diseases has a feature called “focus on flu”

• A detailed report from the US Centers for Disease Control and Prevention titled “Framework for Evaluating Public Health Surveillance Systems for Early Detection of Outbreaks” includes a simple description of syndromic surveillance

• The International Society for Disease Surveillance has a collaborative syndromic surveillance public wiki

• The Anthropology of the Contemporary Research Collaboratory includes working papers and discussions by cultural anthropologists studying modern vital systems security and syndromic surveillance

## Introduction

Throughout the twentieth century, epidemic and pandemic influenza was responsible for causing widespread illness, economic disruption, and considerable loss of life worldwide [1–3]. While the seasonal recurrence of influenza is anticipated each year, it remains difficult to predict the predominant seasonal strains and impossible to know when and where the next human pandemic will emerge. Timely regional monitoring of influenza-related morbidity is a priority for seasonal surveillance and pandemic preparedness [4].

Influenza surveillance currently conducted in the United States encompasses systems for monitoring influenza-related illness and death, investigating unusual respiratory disease outbreaks, and identifying and characterizing viral influenza strains [5]. The rapid epidemiological assessment of influenza-related morbidity and mortality remains a challenge for public health due to the nonspecific symptoms, rarity of laboratory confirmation, and difficulty in obtaining continuous, representative, and age-specific data [1,2]. Across the US, influenza-like illness (ILI) and pneumonia and influenza (P&I) mortality surveillance coordinated by the Centers for Disease Control and Prevention (CDC) has relied on weekly reporting of ILI visits by physicians in a voluntary sentinel network, and of P&I deaths by participating municipal vital records offices [5]. The primary shortcomings of these systems include the burden on health department and physician practices resulting in low and variable participation, with reporting delays and an absence of timely year-round data limiting their usefulness.

In recent years, New York City (NYC) and other jurisdictions have tracked influenza using syndromic surveillance systems such as those based on electronically reported emergency department (ED) patient chief complaints [6]. Several studies based on these systems have reported seasonal increases in respiratory or influenza-like syndrome visits coincident with documented influenza epidemics [6–12]. Coded respiratory and influenza-like ED visits have been reported to provide a timely, sensitive, and year-round measure that enables detection of epidemic influenza 1–2 wk earlier than does P&I mortality data [7]; and age-specific respiratory visits have been reported to show that data from children aged 3–4 y provide the earliest indicator, leading P&I mortality by 7 wk [8]. The absence of viral surveillance data from these studies, however, leave important questions unanswered: To what degree do early seasonal morbidity increases correlate with actual influenza or other respiratory virus circulation? And to what degree does circulating viral type, subtype, and strain impact the timing and magnitude of age-specific morbidity and mortality?

Epidemiologic community and family studies [13–18] and retrospective analyses of mortality and hospitalization data [19–27] have provided considerable insight into the age-specific patterns of seasonal and epidemic influenza. Prospective morbidity surveillance systems, however, have not taken full advantage of age-specific data. Important epidemiologic insights gained by scrutinizing how influenza impacts specific age groups include evidence that influenza often spreads earliest among school-age children [1,16–18]; that school breaks may slow or delay seasonal impact [1,16]; that age-specific impact can be related to prior antigenic exposure in the population [1–3,28]; and that each influenza pandemic last century was marked by a signature shift in relative impact from older to younger age groups [24,25].

The purpose of this study was to evaluate the use of ED visit data for monitoring the age-specific timing and impact of epidemic influenza by predominant circulating viral type, subtype, and antigenic strain. Using a broad definition of ED visits classified as fever or respiratory syndrome chief complaints, we applied a statistical method, routinely used for monitoring clinical ILI [29,30] and P&I mortality data [5,19,20], to ED surveillance data to monitor visits during periods of influenza circulation and provide a surrogate measure of incident influenza-attributable ED visits in NYC. By quantifying and visualizing the temporal and age-specific course of influenza morbidity in the context of available laboratory surveillance data, we sought to improve ongoing influenza surveillance efforts in NYC.

## Methods

### Emergency Department Surveillance Data

Electronic reporting of ED chief complaint data from NYC hospitals occurred daily during the study period from mid-November 2001 through June 2006. Data received each morning were typically >90% complete for the preceding day, and data received Monday mornings >95% complete for the preceding week. During the 2001–2002 season participating hospital EDs captured an estimated 65% of all ED visits citywide. Coverage gradually increased through the study period, reaching 79% of all ED visits citywide during 2002–2003, 88% during 2003–2004 and 90% during 2004–2005 and 2005–2006. Individual ED visit data were aggregated by age group, chief complaint syndrome group, and week ending Saturday.

Of the 13.3 million ED visits reported by participating NYC facilities during the study period, 2.3 million were categorized into a broad “fever and respiratory” syndrome composed of the hierarchical and mutually exclusive syndromes “respiratory,” “fever/flu,” “common cold,” and “sepsis,” as previously described [6]. These syndromes have been used as part of daily surveillance activities in NYC since 2001, and were defined as follows: The “sepsis” syndrome captured ED visits whose chief complaint contained key words representing sepsis, bacteremia, cardiac arrest, unresponsive, unconscious, or dead on arrival—the sepsis syndrome was included to capture visits with chief complaints describing potential, severe influenza outcomes that would otherwise have been missed. The “common cold” syndrome captured visits with key words representing stuffy nose or nasal or cold symptoms that were not in visits captured within sepsis. The “respiratory” syndrome captured visits with key words and International Classification of Diseases 9th edition (ICD-9) codes representing pneumonia, shortness of breath, bronchitis, upper respiratory tract infection, difficulty breathing, pleurisy, croup, cough, dyspnea, and chest cold, which were not captured within the sepsis or common cold syndromes. And the “fever/flu” syndrome captured visits with key words and ICD-9 codes representing fever, chills, malaise, body aches, viral syndrome, and influenza, which were not captured within the sepsis, common cold, or respiratory categories, and did not include key words representing acute gastroenteritis, enteritis, or diarrhea. While chief complaints of “fever with diarrhea” could potentially be due to influenza, these were excluded in our analysis to avoid confounding with coincident epidemic viral gastroenteritis.

**Figure 1:**
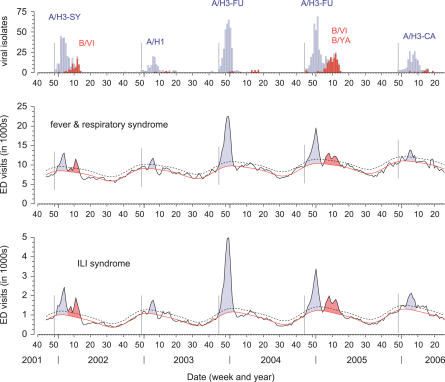
Weekly Influenza Isolates and ED Fever and Respiratory and ILI Visits in New York City during the 2001–2002 to 2005–2006 Seasons Dates are CDC year and week ending Saturday. Top graph, isolates by influenza type are from WHO collaborating laboratories, with subtype and strain designation based on predominant regional and national antigenic lineage: A/H3-SY, predominant circulating A(H3N2) Sydney-lineage viruses; B/VI, predominant circulating B/Victoria-lineage; A/H1, either A(H1N1) New Caledonia- or A(H1N2) Wisconsin-lineage; A/H3-FU, A(H3N2) Fujian-lineage; B/YA, B/Yamagata-lineage; and A/H3-CA, A(H3N2) California-lineage. Middle and bottom graphs, observed fever and respiratory syndrome (middle) and ILI syndrome (bottom) ED visits are shown as black lines, and seasonally expected Serfling baseline visits as red lines. Dashed lines represent epidemic thresholds as model estimates plus two standard deviations. Shaded areas represent estimated influenza-attributable excess ED visits: blue areas correspond to periods of increasing and dominant influenza A circulation and red areas to influenza B. Vertical lines indicate the first week of continuous influenza isolate reporting each season.

**Figure 2:**
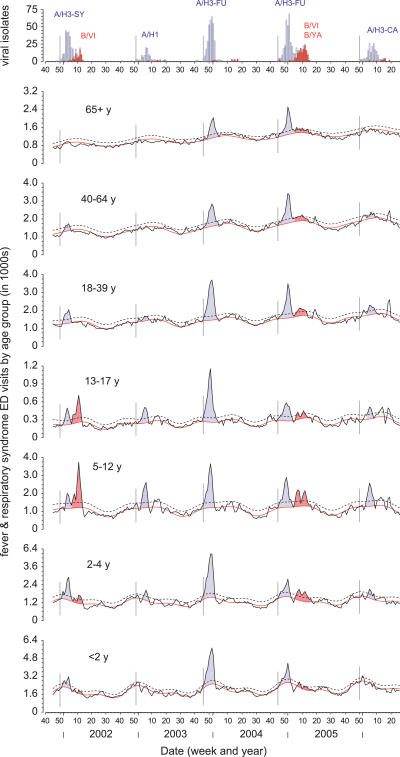
Weekly Age-Specific Fever and Respiratory ED Visits in New York City during the 2001–2002 to 2005–2006 Seasons Observed fever and respiratory ED visits by age group are shown as black lines, and seasonally expected Serfling baseline visits are as red lines. Dashed lines represent model estimates plus two standard deviations. Shaded areas represent influenza-attributable excess ED visits by type A (blue) or B (red). Vertical lines indicate the first week of continuous influenza isolate reporting. Codes in top graph: A/H3-SY, influenza A(H3N2) Sydney; B/VI, influenza B/Victoria; A/H1, either A(H1N1) New Caledonia or A(H1N2) Wisconsin; A/H3-FU, influenza A(H3N2) Fujian; B/YA, influenza B/Yamagata; and A/H3-CA, influenza A(H3N2) California.

The broad “fever and respiratory” syndrome category described above was used to provide the most sensitive measure of ED visits potentially attributable to influenza. We also created a specific “ILI” syndrome following the commonly used clinical surveillance definition of fever with cough and/or sore throat: Of the 2.3 million visits categorized into the broad fever and respiratory syndrome, 260,000 visits were categorized as ILI, defined as a chief complaint composed of an influenza keyword or of a fever-related key word with a mention of “cough” and/or “sore throat.” The broad fever and respiratory and the narrow ILI syndrome data are shown in Figure 1. ED visit data aggregated by age into the groups < 2 y, 2–4 y, 5–12 y, 13–17 y, 18–29 y, 40–64 y and ≥ 65 y, are shown for the broad fever and respiratory group in Figure 2, and the narrow ILI group in Figure S1.

### Laboratory Surveillance Data

Weekly counts of influenza A and Bvirus isolates were reported duringthe study by three World HealthOrganization (WHO) collaboratinglaboratories located in NYC(Figure1). We used the viral isolatedata to identify “influenzacirculation periods,” defined asbeginning the week when one or moreisolates were reported in twoconsecutive weeks leading continuouslyinto the seasonal epidemic period(vertical linesin Figures1, 2, S1, and S2) and ending when the last isolate was reported each season. We defined predominant “influenza epidemic periods” as the consecutive upper quartile weeks of viral isolate reporting during the five influenza seasons in the study: these were simply the worst 25% of weeks, or the 61 weeks reporting the greatest number of viral isolates during the 244-wk study period. National and mid-Atlantic regional influenza subtype and antigenic strain surveillance data reported by CDC [5] were used to infer likely dominant strains circulating during each period of influenza activity in the city (Table 1).

**Table 1:**
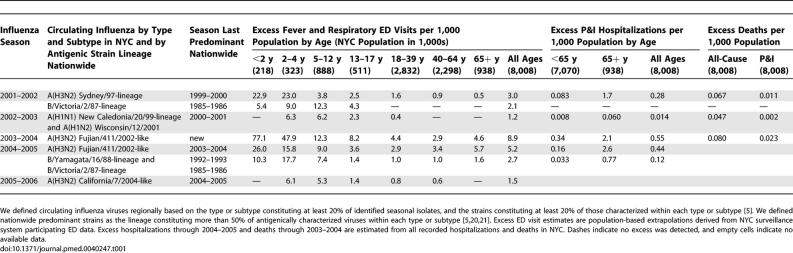
Summary of Excess Emergency Department Visits, Hospitalizations, and Deaths per 1,000 Population in New York City during the 2001–2002 to 2005–2006 Influenza Seasons

### Hospitalization and Death Data

**Figure 3:**
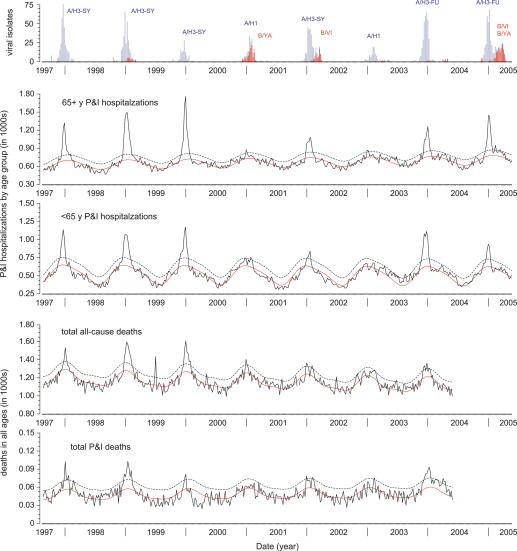
Weekly P&I Hospitalizations and All-Cause and P&I Deaths in New York City Observed P&I hospitalizations by age group from 1998–1999 to 2004–2005, and deaths for all ages from 1998–1999 to 2003–2004, are shown as black lines. Seasonally expected Serfling baseline levels are shown as red lines, and two-standard-deviation thresholds are shown as dashed lines. Catastrophic event deaths were removed from the data, and heat-wave period deaths were censored from the Serfling analysis. Observed P&I deaths during the 1999–2000 season were low due to a changeover from ICD-9 to ICD-10 coding. Codes in top graph: A/H3-SY, influenza A(H3N2) Sydney; B/VI, influenza B/Victoria; A/H1, either A(H1N1) New Caledonia or A(H1N2) Wisconsin; A/H3-FU, influenza A(H3N2) Fujian; B/YA, influenza B/Yamagata; and A/H3-CA, influenza A(H3N2) California.

Confirmed hospitalizations by admission date in NYC from 1997–1998 to 2004–2005 were analyzed by multiple cause ICD-9 code reports for influenza (487), P&I (480–487), and respiratory syncytial virus (RSV) (079.6, 466.11) [22,23]. Confirmed deaths from 1997–1998 to 2003–2004 were analyzed as all-cause and by reported primary cause of death code for P&I (ICD-9 480–487; ICD-10 J10.0–J11.8, J12.0–J18.9) [20,21]. The hospitalization and death data for 1997–1998 through 2000–2001 are shown for comparison (Figure 3). Data were aggregated by week ending Saturday, and by < 65 y and ≥ 65 y age groups.

### Estimating Excess Morbidity and Mortality

Weekly counts of ED visits coded by chief complaint into the broad fever and respiratory syndrome and the narrow ILI syndrome categories followed annual sinusoidal patterns of winter seasonal increase, punctuated by seven distinct periods of 6–12 wk duration coincident with positive influenza A or B isolate reporting by WHO collaborating laboratories in the city (Figure 1). Weekly counts of P&I hospitalizations from 1997 to 2005 and deaths from 1997 to 2004 similarly followed a pattern of winter-seasonal increase, punctuated by influenza A/H3N2 epidemics (Figure 3). To estimate the number of ED visits, hospitalizations, and deaths attributable to influenza we used a traditional Serfling regression approach [5,19,20,29,30] applied to the weekly time series of ED visits, hospitalizations, and deaths, censoring influenza epidemic periods before fitting the model. To calculate expected ED visits for each group we used the equation

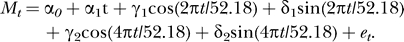



Our estimates of expected ED visits by age and syndrome group during week *t (M_t_)* were derived from least squares regression of the observed data by group using a constant *α_0_,* secular trend *α_1_,* annual sinusoidal terms *γ_1_* and *δ_1_,* semiannual terms *γ_2_* and *δ_2_,* and an error term *e_t_,* applied to noncensored weeks. Estimates of excess weekly ED visits were calculated as observed minus expected. Our significance level, or “epidemic threshold,” was arbitrarily set at two standard deviations above the expected, as derived from the model variance of nonepidemic weeks. We applied the seasonal model and threshold to all weekly ED visit, hospitalization, and death time series.

Estimates of excess seasonal epidemic ED visits were calculated as observed minus expected visits during influenza epidemic periods and consecutive weeks adjacent to those periods when the observed was above expected (shaded areas, Figure 1; Table 1). The nonadjacent periods of significant (i.e., over two standard deviations above Serfling baseline) excess ED visits occurring outside of influenza circulation periods were assumed not to be influenza-attributable and are considered separately, below. Our estimates of total citywide excess ED visits were extrapolated based on the proportion of ED visits citywide that were captured each season by participating hospitals in the surveillance system. Total citywide influenza season excess ED visit rates were calculated based on year 2000 age-specific US Census data for NYC (Table 1).

To better understand age-specific visit patterns that occurred during periods of sporadic or no influenza circulation, we evaluated the timing and impact of two additional causes of seasonal respiratory illness: RSV and tree pollen. We identified predominant RSV periods as the upper quartile weeks of RSV hospitalizations from 2001 to mid-December 2005 (data shown in Figure S2) and predominant seasonal tree pollen periods as the 4 wk of greatest tree pollen counts recorded in 2005 and 2006 from a single environmental monitoring site in NYC.

### Estimating Epidemic Timing

**Figure 4:**
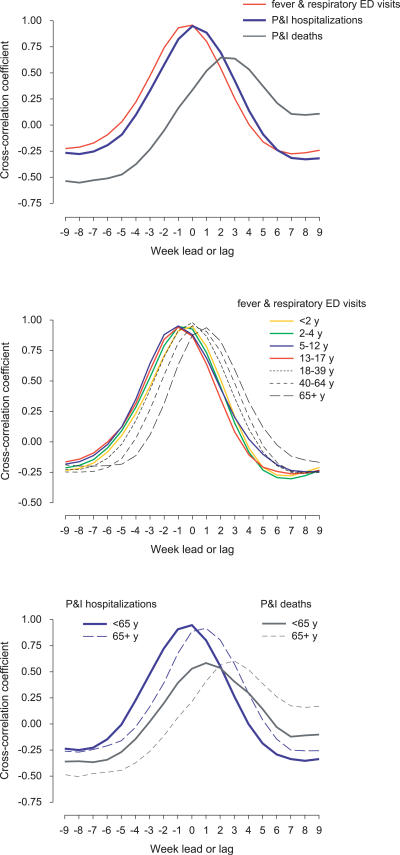
Epidemic Weekly Lead or Lag Cross-Correlation during the 2003–2004 Influenza A/Fujian Season in New York City Excess fever and respiratory ED visits and P&I hospitalizations and deaths were correlated against influenza isolates during the 33 wk centered on peak isolates. Top, maximum cross-correlation values for ED visits and hospitalizations coincided with influenza isolates (no lag) with ED visits leading hospitalizations, by less than 1 wk. Maximum correlation values for P&I deaths lagged isolates by 2 wk. Middle, maximum correlation values for 2–4 y, 5–12 y, and 13–17 y ED visits led isolates by 1 wk, < 2 y, 18–39 y, and 40–64 y visits had no lag, and ≥ 65 y visits lagged isolates by 1 wk. Bottom, maximum cross-correlation values for < 65 y P&I hospitalizations had no lag, for ≥ 65 y hospitalizations and < 65 y P&I deaths had a 1 wk lag, and for ≥ 65 y P&I deaths had a 3 wk lag.

We defined initial detection of annual influenza epidemics as the first week ED visits, hospitalizations or deaths exceeded the two-standard-deviation threshold above model baseline. We compared the date of initial detection based on ED visits, hospitalizations, and deaths by age group (Table S1). We additionally evaluated age-specific epidemic timing by cross correlation [7], restricting our analysis to the 33-wk window centered on the viral influenza isolate peaks each season. We calculated Pearson cross-correlation coefficients with lags. And we considered the lag or lead time with the greatest single week coefficient, or with similar consecutive week coefficients, as providing the best estimated measure of inherent epidemic timing relative to influenza isolates. We evaluated age-specific epidemic timing for ED visits, hospitalizations, and deaths for 2003–2004, the only influenza season in our study with available data and with significant excess ED visits, hospitalizations, and deaths across age groups (Figure 4).

### Visualization of Morbidity by Age

**Figure 5:**
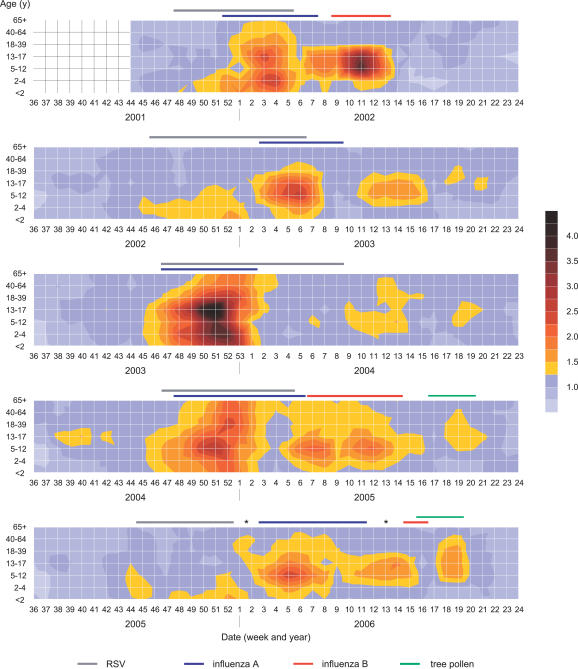
Observed Fever and Respiratory ED Visit Surface Plots by Age Group in New York City during the 2001–2002 to 2005–2006 Influenza Seasons Each season is shown from early September through mid-June by CDC week and year. Weeks of predominant influenza A (blue bar) or B (red bar) isolate surveillance during the study period, retrospectively identified predominant RSV hospitalizations through 2005 (gray bar), and dominant tree pollen periods for 2005 and 2006 (green bar) are shown above each season. Weekly ED visits by age group were detrended and normalized: age-specific intensity is shown as a color gradient interpolated between data points, with observed visits ranging from 2 to >4 times mean noninfluenza levels during peak epidemic weeks, and 0.25 to 1.25 during nonepidemic periods. Visits were increased across all age groups during periods of influenza A/H3N2 predominance, and were most markedly increased during the 2003–2004 and 2004–2005 A/Fujian-lineage epidemics. Visit increases during periods of influenza A/H1 and B predominance impacted preschool (2–4 y) and school-aged (5–17 y) children, and were most dramatically elevated during the B/Victoria-lineage reemergence in early 2002. The autumn and early-winter predominance of RSV preceded influenza in 2001, 2002, and 2005, and coincided with increased visits in the < 2 y and 2–4 y age groups. Visits were notably increased among school-aged children and working-aged adults during the dominant tree pollen period in 2006. RSV hospitalization data were not available for 2006 (*), and viral influenza surveillance reporting was incomplete during weeks 12 to 16, 2006 (*).

To visualize the temporal course of age-specific illness trends in NYC, fever and respiratory ED visits were detrended, normalized, and plotted by week and age group. To detrend the data, we fit a least-squares linear regression to the nonepidemic fever and respiratory ED visit data by age group, as above, but with the annual and semiannual seasonal sinusoidal terms removed. We divided the observed data time series by the nonepidemic linear fit to obtain normalized ED visits by week and category. We made a surface plot of normalized weekly time series as a gradient interpolated between adjacent week and age-group data points (Figure 5). Influenza epidemic periods, based on WHO collaborating laboratory isolate reports in NYC, from 2001–2002 to 2005–2006 were shown above each seasonal plot, and seasonal RSV predominant periods were shown as the upper quartile weeks of RSV hospitalizations from 2001 through 2005, based on the available data for all ICD-9 coded hospitalizations in NYC (Figures 5 and S2). Dominant tree pollen periods, based on available data from a single monitoring site in NYC, were shown above the spring 2005 and 2006 plots (Figure 5).

## Results

### Seasonal Impact

From the 2001–2002 to 2005–2006 influenza seasons in NYC, we estimate on average that 40,000 excess ED visits (5.0 visits per 1,000 population) occurred per season during the documented influenza circulation periods. We estimate that 2,800 excess P&I hospitalizations (0.35 per 1,000) on average occurred per season from 2001–2002 to 2004–2005, and 500 excess all-cause (0.065 per 1,000) and 100 excess P&I (0.012 per 1,000) deaths occurred per season from 2001–2002 to 2003–2004. The seasonal impact of excess ED visits, hospitalizations, and deaths, however, varied greatly by age group and circulating virus. We summarize our results by season and predominant viral period.

2001–2002 season: An estimated 24,000 excess fever and respiratory ED visits (3.0 visits per 1,000 population), 2,200 excess P&I hospitalizations (0.28 per 1,000), 540 excess all-cause deaths (0.067 per 1,000), and 90 excess P&I deaths (0.011 per 1,000) occurred during the influenza A/H3N2 predominant period in NYC from December 2001 to February 2002 (weeks 50–07) (Figures 1–3; Table 1). An estimated 17,000 excess ED visits (2.1 per 1,000) occurred during the influenza B/Victoria predominant period from February to April 2006 (weeks 08–13) (Figure 1; Table 1). Excess ED visits during the influenza B/Victoria-period were most notably increased among school-aged children (aged 5–12 y and 13–17 y), with no detected increase among adults (Figures 2 and 5; Table 1). There were no excess P&I hospitalizations or deaths detected during the influenza B period this season (Figure 3).

2002–2003 season: An estimated 10,000 excess ED visits (1.2 per 1,000 population) occurred during the predominant influenza A/H1 period (Figure 1; Table 1), with excess visits detected only among preschool (2–4 y) and school-aged (5–12 y and 13–17 y) children, and adults aged 18–39 y (Figure 2). Visits among children age < 2 y and 2–4 y were increased during autumn and early winter (weeks 45–01) prior to the influenza epidemic period (weeks 03–09). The autumn and early winter timing of ED visit increases among children age <2 y and 2–4 y (weeks 45–01) coincided with the retrospectively identified period of predominant RSV hospitalizations (Figures 2 and S2). An estimated 370 excess all-cause deaths (0.047 per 1,000), and few excess P&I hospitalizations or deaths, were detected during this season (Figure 3; Table 1).

2003–2004 season: An estimated 71,000 excess fever and respiratory ED visits (8.9 per 1,000 population) occurred during the influenza A/H3N2 predominant period from November 2003 to January 2004 (weeks 46–01) (Figure 1; Table 1). Excess ED visits were detected across age groups (Table 1), with the highest rates occurring among children age <2 y (Figures 2 and 5; Table 1). An estimated 4,400 excess P&I hospitalizations (0.55 per 1,000), 640 excess all-cause deaths (0.080 per 1,000), and 190 excess P&I deaths (0.023 per 1,000) occurred this season (Figure 3; Table 1).

2004–2005 season: An estimated 42,000 excess fever and respiratory ED visits (5.2 per 1,000 population) and 3,600 excess P&I hospitalizations (0.44 per 1,000) occurred during the influenza A/H3N2 predominant period from November 2004 through January 2005 (weeks 46–04) (Figures 1 and 3; Table 1). An estimated 22,000 excess ED visits (2.7 per 1,000) occurred during the period of influenza B predominance and sporadic influenza A circulation from February to April 2005 (weeks 05–14) (Table 1). Excess ED visits were increased across all age groups during the influenza A-predominant period (Figures 1 and 2). Compared to the 2003–2004 epidemic, the relative impact during the corresponding A/H3N2 epidemic weeks in 2004–2005 was shifted toward older age groups: the proportion of total excess fever and respiratory ED visits among those ≥ 65 y went from 6% in 2003–2004 to 13% in 2004–2005, and the proportion of total excess P&I hospitalizations among those ≥ 65 y went from 45% in 2003–2004 to 69% in 2004–2005 (Figure 2; Table 1).

2005–2006 season: An estimated 12,000 excess ED visits (1.5 per 1,000 population) occurred during the influenza A/H3N2 predominant period, with excess ED visits detected in the age groups 2–4 y, 5–12 y, 13–17 y, 18–39 y, and 40–64 y (Figure 2; Table 1). Fever and respiratory ED visits peaked in the < 2 y age group prior to the beginning of the identified influenza epidemic period and more than 4 wk before the peak in influenza isolate data (Figure 2).

### Epidemic Timing

Influenza epidemic period increases were seen earlier in ED visits than in hospitalizations or deaths. During the influenza A/H3N2 epidemics in 2001–2002 and 2003–2004, excess all-ages fever and respiratory ED visits exceeded our two-standard-deviation Serfling model threshold 1 wk prior to P&I hospitalizations and, respectively, 1 and 3 wk prior to P&I deaths. During the mild influenza A/H1 epidemic in 2002–2003, all-ages fever and respiratory ED visits exceeded threshold 2 wk prior to deaths, and in the A/H3N2 epidemic in 2004–2005, all-ages ED visits exceeded threshold 3 wk prior to P&I hospitalizations (Figures 1 and 3; Table S1).

Fever and respiratory ED visits among children often exceeded threshold before adults, but there were differences between seasons. During 2001–2002, ED visits exceeded model thresholds in the < 2 y and 2–4 y age groups 1 wk before the 13–17 y and 18–39 y age groups, and 2 wk before the 5–12 y and 40–64 y age groups. During the more severe 2003–2004 A/H3N2 epidemic, age-specific ED visits exceeded threshold in the 13–17 y age group 1 wk before the < 2 y, 2–4 y, 5–12 y, and 18–39 y groups, 3 wk before the 40–64 y group, and 4 wk before the ≥ 65 y group (Figure 2; Table S1). During the 2004–2005 influenza A/H3N2 epidemic, age-specific ED visits exceeded model threshold in the 2–4 y, 5–12 y, and 13–17 y age groups 1 wk prior to the < 2 y, 18–39 y, 40–64 y, and ≥ 65 y age groups (Figure 2; Table S1). The threshold level was arbitrary, with the measure of timing reflecting noninfluenza period variance and not necessarily inherent epidemic timing.

In our estimation of inherent epidemic timing, we limited our analysis to the 2003–2004 season, since it was the most severe and the only one with available ED, hospitalization, and death data and significant excess estimates across age groups (Figures 1–3). Our lagged cross-correlation analysis of excess ED visits, hospitalizations, and deaths compared against viral influenza surveillance data found the greatest cross-correlation coefficients occurred when P&I deaths lagged viral data by 2 wk, and P&I hospitalizations and ED visits coincided with viral data, although the leading cross-correlation coefficients for ED visits were greater than for hospitalizations (Figure 4, top). The maximum cross-correlation coefficient for viral isolates and excess ED fever and respiratory visits by age found school-aged children (5–12 y and 13–17 y) leading viral isolates by 1 wk, preschool-aged children (2–4 y) leading viral isolates by 1 wk although lagging school-aged children slightly, younger children (<2 y) and working-aged adults (18–39 y and 40–64 y) roughly coinciding with viral isolates, and older adults (≥65 y) lagging viral isolates by 1 wk (Figure 4, middle). The maximum cross-correlation coefficient for P&I hospitalizations and deaths by age group found < 65 y hospitalizations coinciding with viral isolates, ≥ 65 y hospitalizations and < 65 y P&I deaths lagging viral isolates by 1 wk, and ≥ 65 y deaths lagging viral isolates by 3 wk (Figure 4, bottom).

### Visualizing Age-Specific Morbidity Patterns

Observed fever and respiratory ED visits peaked annually during influenza epidemic periods as defined by laboratory evidence (Figure 1). The age pattern of observed and normalized visits varied by season and circulating virus (Figures 2 and 5). Each autumn, increased visits were seen among children aged < 2 y and 2–4 y during RSV-predominant periods, and were notably elevated between autumn and early-winter (weeks 48–01), regardless of whether influenza circulation was detected (Figure 5): for example, seasonal fever and respiratory ED visit peaks in the < 2 y age group occurred during week 52 in 2002–2003 and 2005–2006, prior to the beginning of the defined influenza epidemic period and well before viral influenza isolates and older-age group ED visits peaked (Figures 2 and 5). Otherwise, during each influenza epidemic period in our study, fever and respiratory ED visits peaked earliest in school-aged children. The ED visit peaks in the 5–12 y and 13–17 y age groups during the two most severe seasons, 2003–2004 and 2004–2005, immediately preceded the end-of-year winter holiday school breaks.

The more specific subset of ILI ED visits, with mention of influenza or co-occurrence of fever with cough and/or sore throat, constituted only 11% of the broader fever and respiratory category (Figure 1), however the two trends were highly correlated (*r* ^2^ = 0.96, *p* < 0.001). Exceptions included tree pollen-dominant periods in spring 2005 and 2006 when the broad fever and respiratory category of ED visits saw increases among school-aged children and working-aged adults (5–12 y, 13–17 y, 18–39 y, and 40–64 y) (Figure 5), with no corresponding increase in the more specific ILI category (Figures 2 and S1).

## Discussion

In our analysis of New York City ED data, we found that predominant increases in fever and respiratory visits corresponded in timing and magnitude with laboratory-confirmed influenza, and we suggest that our estimates of excess ED visits provide a reliable surrogate measure of the incident impact attributable to influenza. By applying standard statistical methods to electronic ED chief complaint data, and interpreting results in the context of available information about circulating viruses, we were able to evaluate and track age-specific influenza morbidity in greater detail than was previously possible in NYC. We found the burden of excess ED visits was greatest during peak influenza periods, disproportionately impacted children, often impacted children earliest, generally coincided in timing with P&I hospitalization admission data, and preceded P&I death data by roughly 1–2 wk. The age-specific pattern of excess ED visits varied depending on the predominant circulating viral type, subtype, and strain. We expand on these findings below.

### Reemergence of B/Victoria-Lineage Influenza, 2001–2002

Beginning the week ending February 16, 2002 (week 06–2002), a marked and sustained increase in ED fever and respiratory visits began in NYC that predominated among school-aged children (5–17 y). In the US, influenza B/Victoria-lineage viruses had last been widespread 13 y prior, during 1988–1989 in a mixed influenza B and A/H1N1 season, and had last been the predominant epidemic virus 16 y earlier, during the 1985–1986 season [23,31,32]. This pattern would suggest that in 2002, children age 13–16 y had minimal prior exposure, and children age 12 y and under had very little or no opportunity for prior exposure to this influenza B antigenic lineage, consistent with observed excess fever and respiratory (Figure 2) and ILI (Figure S1) ED visits. Local clinical and outbreak reports later noted the unusual age-specific impact of these B/Victoria viruses elsewhere in the US [33,34], and school absenteeism and ED surveillance in NYC were noted to have signaled significant increases during the epidemic [6,35]. However, ongoing morbidity surveillance at the time did not detect or characterize the impact [5,6], and no comment was made connecting previous circulation of this antigenic lineage with the age-specific groups at risk. A greater awareness of the event through routine monitoring of age-specific illness data could have informed physicians and public health officials that an influenza virus antigenically novel to children age 12 y and under was epidemic.

### Epidemic A/H3N2 Fujian-Lineage Influenza

Antigenic variant influenza A/H3N2 Fujian-lineage viruses emerged in autumn 2003 and were widespread across the US by the beginning of winter. The 2003–2004 seasonal influenza vaccine was reported to be poorly matched with the circulating A/Fujian viruses [5]. Our analysis of fever and respiratory ED visits indicated that the epidemic impact on morbidity was significant across all age groups, though greater among children (Figure 2; Table 1) and with a distinct within-season age shift (Figure 4). Our analysis of the timing of ED visits indicates that the course of the epidemic progressed in a cascading fashion from primary school (5–17 y), to preschool (2–4 y), to younger children and working-age adults (<2 y and 18–64 y), to older adults (≥65 y) (Figure 4). Our analysis of ED visits and P&I hospitalizations and deaths indicates that the morbidity impact seen in ED visits and hospitalizations preceded the impact seen in deaths by 1–2 wk, with P&I hospitalizations and deaths following a similar age-stratified progression from younger (<65 y) to older (≥65 y) ages (Figure 4).

In autumn 2004, influenza viruses reported to be antigenically A/Fujian-like [5] reemerged and were epidemic in NYC. The pattern and age distribution of morbidity in 2004–2005 presented a distinct shift in impact compared to 2003–2004, in both ED fever and respiratory and ILI visits (Figures 2 and S1) and P&I hospitalizations (Figure 3). The relative impact in children and younger adults was greater during the first reported circulation of A/Fujian-lineage viruses in NYC, while the relative impact in older adults was greater during the second (Table 1). It is not known if the age shift from 2003–2004 to 2004–2005 was due to genetic or antigenic differences in the circulating viruses, or to age-specific cohort effects in transmission and impact. It is also not known whether this interpandemic period shift was similar in character to the shift in impact to older adults seen in the successive seasons following primary pandemic waves in the last century [1,24].

### Seasonal RSV

While RSV surveillance data were not available during the study period, coded hospitalizations allowed us to retrospectively identify predominant RSV periods in NYC (Figure S2). The impact of RSV hospitalizations during the 2001–2002, 2002–2003, and 2005–2006 seasons occurred prior to the beginning of the influenza epidemic periods (Figure 5). During these seasons, increases in fever and respiratory ED visits occurred in the age groups < 5 y before documented influenza circulation began. Across all seasons the course of fever and respiratory ED visits in young children increased by mid-November, regardless of the timing or impact of influenza. The absence of a significant increase in ED visits among school-aged children or adults during these periods was consistent with RSV only modestly impacting older ages, and suggests that estimates of severe excess RSV-attributable mortality in older individuals [23] be reevaluated [36–38]. The age-specific impact on morbidity from RSV and other respiratory infections such as metapneumovirus [39] or novel rhinoviruses [40], should be considered when evaluating fever and respiratory morbidity surveillance and should be studied further.

### Spring Tree Pollenosis

In NYC we have consistently observed increases in ED respiratory and asthma visits outside of influenza season during the spring and early fall. The impact seen each spring is often severe enough to affect any syndrome that includes respiratory chief complaints, but is not associated with an increase in febrile illness. Pollen data obtained for the spring of 2005 and 2006 show that increases apparent in the broad fever and respiratory syndrome group among patients aged 5–64 y were coincident with the predominant annual tree pollen release (Figures 2 and 5). A similar pattern of respiratory and asthma exacerbations associated with tree and grass pollenosis has recently been reported [41], and the age-specific pattern of morbidity due to cedar tree pollenosis has been well described in Japan [42].

### Influenza Timing

In our analysis, we found that increases in influenza-attributable ED visits preceded hospitalizations, which in turn preceded deaths (corresponding to the logical progression of illness). During the 2003–2004 A/Fujian epidemic season we found fever and respiratory ED visits and P&I hospitalizations and deaths strongly correlated with viral isolate data, with an optimum lag between ED visits and deaths on the order of 2–3 wk (Figure 4, top). Our age-specific analysis of this epidemic found ED visits by age strongly correlated with viral data, with the greatest lag found between the 5–17 y and ≥65 y age groups on the order of 2 wk, with younger children and working-age adults in between (Figure 4, middle). The time series of P&I hospitalizations by age group were strongly correlated with viral data, with a lag between < 65 y and ≥ 65 y hospitalizations on the order of 1 wk (Figure 4, bottom). The time series of P&I deaths by age group were most strongly correlated with a lag between < 65 y and ≥ 65 y deaths on the order of 2 wk. While we found no strict temporal age pattern across seasons, we did find late autumn increases in ED fever and respiratory visits among children age < 5 y regardless of influenza circulation (and coincident with predominant RSV), and we found that influenza epidemic period peaks occurred earliest among school-aged children each season regardless of circulating influenza viral type, subtype or strain.

A study of Boston area ED surveillance data reported that ED respiratory visits increased first among preschool age children (aged 3–4 y), some 5–7 wk before ED visits among older persons [8]. This finding may have been be due to the impact of RSV, and to the use of aggregate interseasonal waves masking within-season variation by age. Analysis of the period from 2001 to 2006 using these methods on NYC ED visit data would show the age-specific impact of RSV shifting the overall timing of < 5 y ED visits earlier, while the spring influenza B/Victoria epidemic would shift 5–17 y ED visits later. Aggregate interseasonal time series analysis can be valid for seasonal influenza mortality, where a single wave of mortality predominates each season. Assessment of aggregated seasonal time series of ILI, fever, or respiratory morbidity data, where multiple etiologically distinct within-season waves are common, however, must be done with caution and at the appropriate scale [43].

### Influenza Impact

While the burden of influenza-attributable hospitalizations and deaths occurred predominantly among older adults, the burden of influenza-attributable excess fever and respiratory visits to NYC EDs during our study was predominantly among children (Figure 2; Table 1). Our estimated NYC-wide influenza attributable ED visit rates among young children (aged <5 y), during the 2003–2004 A/H3N2-Fujian epidemic, were greater than the ED visit rate estimates reported by Poehling and colleagues [44], and less than their outpatient clinic estimates, possibly reflecting greater utilization of EDs for pediatric primary care in NYC. While the overall impact from influenza during the study period was moderate compared to preceding A/H3N2 Sydney-lineage epidemic seasons (Figure 3), the impact seen in NYC EDs was nonetheless considerable and varied. For each estimated excess P&I death we found during the 2003–2004 A/Fujian epidemic, there were approximately 3.5 excess all-cause deaths, 24 excess P&I hospitalizations, and 390 excess fever and respiratory ED visits in NYC.

Our study had several limitations. First, estimating influenza-attributable morbidity and mortality is imperfect due to the nonspecific nature of influenza symptoms and the lack of laboratory confirmation for the vast majority of influenza cases [1]. We considered excess visits as primarily attributable to influenza when they occurred during periods of virally confirmed influenza circulation, but some of the fever and respiratory syndrome visits outside of these periods were likely due to influenza infection, and to some extent excess visits during influenza periods could clearly be due to coincidentally circulating viruses. Furthermore, the free-text chief complaints used to categorize ED visits into syndrome groups are imprecise indicators of illness, and many influenza-attributable visits may have been missed. We also did not explicitly consider the influence of ED utilization on age-specific visit rates. For example, parents may be more likely to bring a young child to the ED for an evaluation of influenza-like illness than to visit the ED themselves. A greater proportion of younger patients with acute influenza infection may have had fever with respiratory symptoms and been captured in our syndrome definition, while older patients with influenza-attributable illness and complications may have presented later and with a broader range of complaints, many of which might not have been captured by our syndrome coding. Finally, we had only 5 y of data covering a unique, large, and dense urban population, and our findings may not be generalizable to other years or to other regions. Within the context of these limitations, our results highlight the fact that each influenza season and epidemic period is unique in its age-specific timing, progression, and impact.

The reliance of researchers on hospitalization and death data, and the difficulty of obtaining population-based and age-detailed estimates of morbidity have contributed to the misconception that influenza affects only the very young and the very old. While the burden of severe morbidity and mortality occurs at the extremes of age, our findings support the observation that school-aged children experience early and high attack rates and exhibit significant morbidity, supporting evidence that they play an important role in communitywide transmission [16–18]. Vaccination of school-aged children has been suggested to provide both direct protection for those vaccinated and indirect protection for unvaccinated age groups within the population during interpandemic [45] as well as pandemic periods [13]. The early and specific increases in fever and respiratory ED visits that we observed among children during the first season of A/H3N2 Fujian circulation are consistent with other studies showing that epidemic influenza strains may circulate and amplify first among children before spreading to older age groups [16–18]. These findings may have implications for targeted nonpharmaceutical intervention, antiviral, and vaccination strategies, and lend support for broadening the age categories recommended for routine and pandemic vaccination.

Twentieth-century influenza can inform twenty-first-century surveillance. The experience with pandemic influenza in the last century in New York City illustrates that early waves, multiple waves, and within- and between-season age shifts in morbidity and mortality can occur [46–48]. While the timing and age-specific impact in the next pandemic cannot currently be predicted, experience during the last five seasons in New York City suggests that age-stratified ED surveillance can provide a timely surrogate measure of morbidity that can be used to monitor and describe the age-specific epidemiology of influenza. Recent analyses of the 1918 pandemic in US cities have shown that even transitory and imperfect public health intervention strategies, when initiated early enough, were partially beneficial [49,50]. While our study does not identify surveillance triggers for public health intervention or address control measure efficacy, our data do show that near-time monitoring is feasible. Our results highlight the fact that influenza epidemics can differ in timing, progression, and impact by age. The integration of detailed and rapid morbidity surveillance data, such as we have presented, with viral, antigenic, and whole-genome analysis [51–54], may improve our understanding of the complex dynamics of influenza [55], and provide better opportunity for informed and successful public health responses in the future.

## Supporting Information

Figure S1 Weekly Age-Specific ILI Visits to the ED in New York City during the 2001–2002 to 2005–2006 Seasons Observed ILI syndrome ED visits by age group are shown as black lines, and seasonally expected Serfling baseline visits as red lines. Dashed lines represent epidemic thresholds as model estimates plus two-standard deviations. Shaded areas represent estimated influenza attributable excess ED visits: blue areas correspond to periods of increasing and dominant influenza A circulation and red areas to influenza B. Vertical lines indicate the first week of continuous influenza isolate reporting each season.
(249 KB PDF)

Figure S2 Weekly Influenza Viral Isolate Surveillance and RSV Hospitalizations in New York City during the 2001–2002 to 2005–2006 Seasons Vertical lines indicate the first week of continuous influenza virus isolate reporting, viral isolate surveillance is indicated as in Figure 1. The dashed horizontal lines indicate the level of influenza viral isolate and RSV hospitalization upper quartile weeks.
(172 KB PDF)

Table S1 Summary of Consecutive Weeks of Influenza Circulation Reporting, Epidemic Influenza Isolate Reporting, Epidemic Fever and Respiratory ED Visits, and Epidemic P&I Hospitalizations and Deaths in New York City during the 2001–2002 to 2005–2006 Seasons Influenza isolate circulation dates are the CDC weeks from the first influenza isolate reported in continuous weeks (vertical lines in Figures 1, 2, S1, and S2), through the last week reporting an influenza isolate that season. Influenza epidemic weeks represent the upper quartile weeks of influenza isolate reporting (ie the worst 25% of weeks, shown in Figure 5). Epidemic fever and respiratory ED visits (Figures 1 and 2), and P&I hospitalizations and deaths (Figure 3) indicate the continuous weeks exceeding a two-standard-deviation threshold above each Serfling baseline during the periods of influenza circulation. Asterisks indicate continuous weeks that exceeded threshold prior to the beginning of the influenza viral isolate epidemic weeks. Dashes indicate seasons and groups where there were no continuous weeks exceeding threshold. Empty cells indicate no available data.
(36 KB DOC)

## Abbreviations

CDC: Centers for Disease Control and Prevention
ED: emergency department
ILI: influenza-like illness
NYC: New York City
P&I: pneumonia and influenza
RSV: respiratory syncytial virus
