# Supplementary material for: Monitoring the Impact of Influenza by Age: Emergency Department Fever and Respiratory Complaint Surveillance in New York City
Source: PLoS Med. 2007 Aug 7;4(8):e247. doi: 10.1371/journal.pmed.0040247 (PMC1939858; doi:10.1371/journal.pmed.0040247)
Supplement: Supplementary file 1 [file pmed.0040247.sg001.pdf]

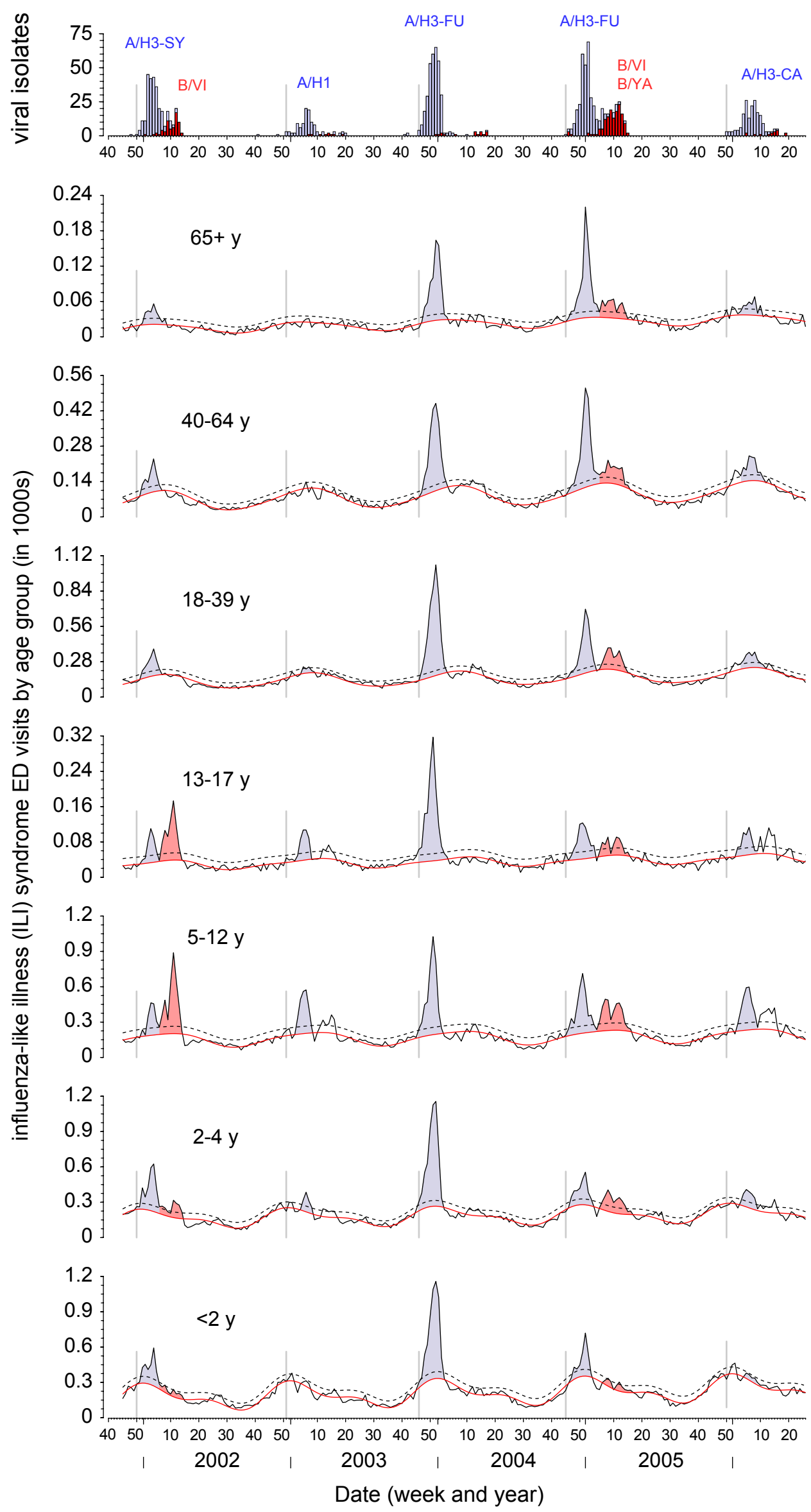
viral isolates
A/H3-SY
B/VI
A/H1
A/H3-FU
A/H3-FU
B/VI
B/YA
A/H3-CA
65+ y
40-64 y
18-39 y
13-17 y
5-12 y
2-4 y
<2 y
influenza-like illness (ILI) syndrome ED visits by age group (in 1000s)
2002
2003
2004
2005
Date (week and year)
