# Supplementary material for: Monitoring the Impact of Influenza by Age: Emergency Department Fever and Respiratory Complaint Surveillance in New York City
Source: PLoS Med. 2007 Aug 7;4(8):e247. doi: 10.1371/journal.pmed.0040247 (PMC1939858; doi:10.1371/journal.pmed.0040247)
Supplement: Supplementary file 2 [file pmed.0040247.sg002.pdf]

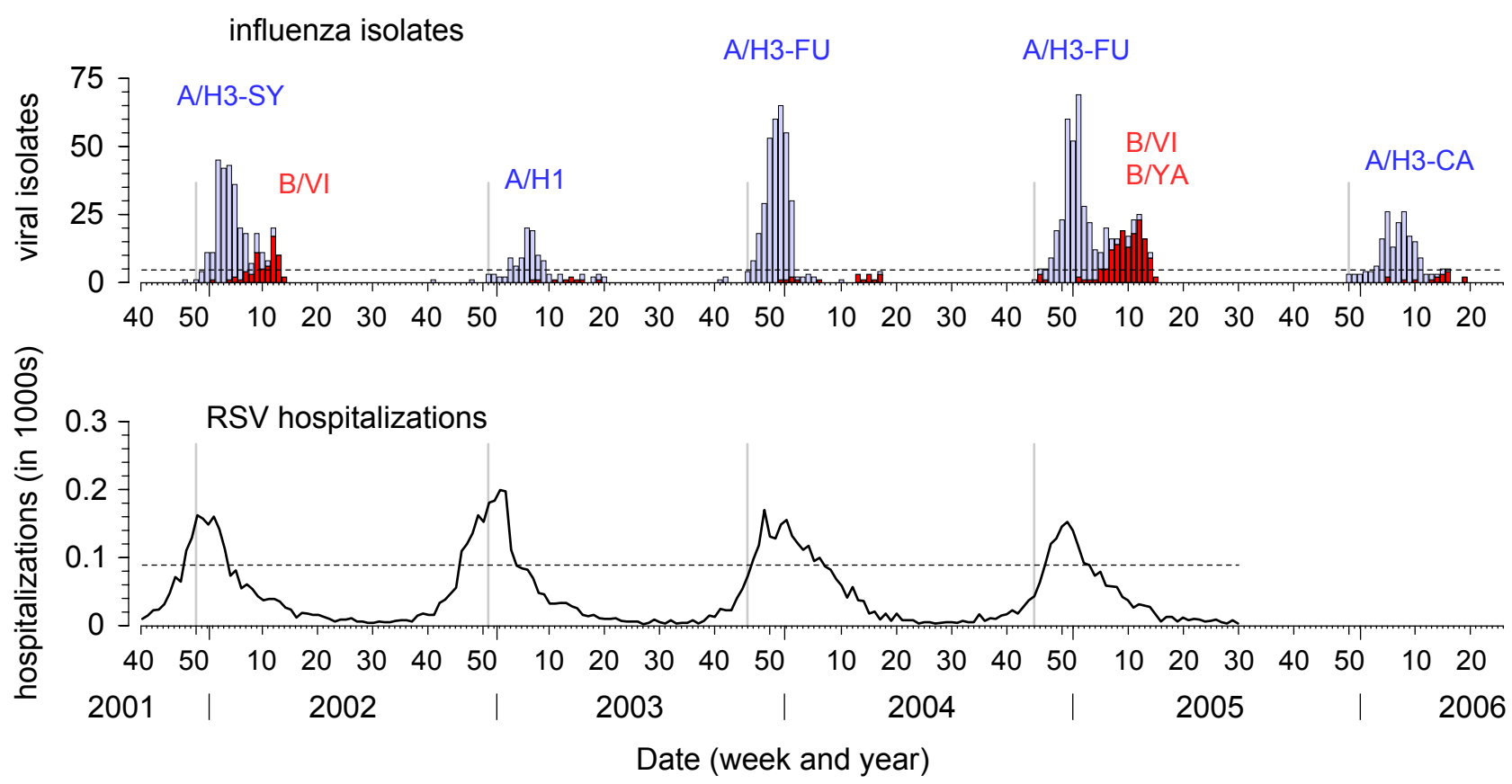

Supporting Figure 2
