# Supplementary material for: Monitoring the Impact of Influenza by Age: Emergency Department Fever and Respiratory Complaint Surveillance in New York City
Source: PLoS Med. 2007 Aug 7;4(8):e247. doi: 10.1371/journal.pmed.0040247 (PMC1939858; doi:10.1371/journal.pmed.0040247)
Supplement: Supplementary file 3 [file pmed.0040247.st001.doc]

**Supporting Table: Summary of consecutive weeks of influenza circulation reporting, epidemic influenza isolate reporting, epidemic fever and respiratory emergency department (ED) visits, and epidemic pneumonia and influenza (P&I) hospitalizations and deaths** in New York City during the 2001-2002 to 2005-2006 seasons.

| Influenza season | influenza viral isolate | | epidemic fever & respiratory ED visit weeks (above 2 sd threshold), by age group | | | | | | | | epidemic P&I hospitalization weeks, all ages | | | epidemic P&I death weeks, all ages | | |
| --- | --- | --- | --- | --- | --- | --- | --- | --- | --- | --- | --- | --- | --- | --- | --- | --- |
|  | circulation weeks | epidemic weeks | <2 y | 2-4 y | 5-12 y | 13-17 y | 18-39 y | 40-64 y | 65+ y | all ages | <65 y | 65+ y | all ages | <65 y | 65+ y | all ages |
| 2001-2002 | 50-14 | 52-13 | 52-05 | 02-05  11-13 | 02-05  07-13 | 02-05  07-12 | 01-05 | 02-04 | - | 52-05  10-12 | 02-04 | 01-06 | 01-05 | - | - | 06-07 |
| 2002-2003 | 51-20 | 03-09 | - | 05-07 | 03-07  14-15 | 03-07  14-15 | 05-06 | - | - | 04-07 | - | 01-02* | - | - | - | - |
| 2003-2004 | 46-17 | 47-01 | 47-01 | 47-53 | 47-53 | 46-01 | 47-01 | 49-02 | 50-02 | 47-01 | 48-01 | 50-03 | 49-01 | 50-02 | 51-03 | 51-03 |
| 2004-2005 | 45-15 | 48-14 | 48-01 | 48-01  06-14 | 48-02  05-08  10-13 | 47-02  06-07  10-11 | 48-03  06-12 | 48-02  09-10 | 48-04 | 47-02  06-13 | 51-01 | 50-03 | 50-02 |  |  |  |
| 2005-2006 | 50-19 | 03-16 | 52-01* | 05-06 | 03-08  11-15 | 04-07  12-14 | 04-08 | 01-02* | - | 04-08 |  |  |  |  |  |  |

Influenza isolate circulation dates are the CDC weeks from the first influenza isolate reported in continuous weeks (vertical lines in Figures 1 and 2 and Supporting Figures 1 and 2), through the last week reporting an influenza isolate that season. Influenza epidemic weeks represent the upper quartile weeks of influenza isolate reporting (ie the worst 25% of weeks, shown in Figure 5). Epidemic fever and respiratory ED visits (Figures 1 and 2), and P&I hospitalizations and deaths (Figure 3) indicate the continuous weeks exceeding a 2 standard deviation threshold above each Serfling baseline during the periods of influenza circulation. Asterisks (*) indicate continuous weeks which exceeded threshold prior to the beginning of the influenza viral isolate epidemic weeks. Dashes indicate seasons and groups where there were no continuous weeks exceeding threshold. Empty cells indicate no available data.
